# Herbarium data: Global biodiversity and societal botanical needs for novel research

**DOI:** 10.1002/aps3.1024

**Published:** 2018-02-28

**Authors:** Shelley A. James, Pamela S. Soltis, Lee Belbin, Arthur D. Chapman, Gil Nelson, Deborah L. Paul, Matthew Collins

**Affiliations:** ^1^ National Herbarium of New South Wales Royal Botanic Gardens and Domain Trust Mrs Macquaries Road Sydney New South Wales 2000 Australia; ^2^ Florida Museum of Natural History University of Florida Gainesville Florida 32611 USA; ^3^ Atlas of Living Australia CSIRO Clunies Ross Street Acton Australia Capital Territory 2601 Australia; ^4^ Australian Biodiversity Information Services Ballan Victoria 3342 Australia; ^5^ iDigBio Florida State University Tallahassee Florida 32306 USA; ^6^ Advanced Computing and Information Systems University of Florida Gainesville Florida 32611 USA

**Keywords:** biodiversity data, biodiversity standards, global change, herbarium collections, informatics

## Abstract

Building on centuries of research based on herbarium specimens gathered through time and around the globe, a new era of discovery, synthesis, and prediction using digitized collections data has begun. This paper provides an overview of how aggregated, open access botanical and associated biological, environmental, and ecological data sets, from genes to the ecosystem, can be used to document the impacts of global change on communities, organisms, and society; predict future impacts; and help to drive the remediation of change. Advocacy for botanical collections and their expansion is needed, including ongoing digitization and online publishing. The addition of non‐traditional digitized data fields, user annotation capability, and born‐digital field data collection enables the rapid access of rich, digitally available data sets for research, education, informed decision‐making, and other scholarly and creative activities. Researchers are receiving enormous benefits from data aggregators including the Global Biodiversity Information Facility (GBIF), Integrated Digitized Biocollections (iDigBio), the Atlas of Living Australia (ALA), and the Biodiversity Heritage Library (BHL), but effective collaboration around data infrastructures is needed when working with large and disparate data sets. Tools for data discovery, visualization, analysis, and skills training are increasingly important for inspiring novel research that improves the intrinsic value of physical and digital botanical collections.

Botanical collecting during the past 450 years has resulted in the permanent housing of more than 381 million specimens in over 3000 herbaria scattered across the globe (Krishtalka et al., 2016; Thiers, 2017). Such collections continue to be gathered and are a valuable record of biodiversity across biomes and through time, from deep time to yesterday (Gardner et al., 2014; Page et al., 2015; Holmes et al., 2016; Willis et al., 2017). The increasing challenges of funding highlight the need for herbaria and botanical collections to improve curation efficiency. With ever‐improving digital technologies enhancing database and image‐capture workflow efficiencies, the complete cataloging of collections is now possible. Collections data can be published and shared with data aggregators, allowing for global discovery, synthesis, and prediction. This paper provides an overview of how aggregated, open access botanical and associated genetic, trait, environmental, and ecological data sets are available for assessing the impacts of global change on communities, organisms, and society; predicting future impacts; and helping to drive the remediation of change. The challenges of using such data are discussed.

## Research use of herbarium data

Herbarium specimens and their data are, for the most part, verifiable, repeatable, sustainable, and persistent (Page et al., 2015; Holmes et al., 2016). Temporal data across taxonomic groups, communities, and habitats enable assessment of changes in species distributions, dispersal ability, or clade differences. Interactions within and between taxa can be interpreted, providing information about species associations and community assemblages through space and time. Historical and reliable baseline data from collections are needed to build robust predictive models for various taxon‐level or functional‐group global change responses (e.g., Willis et al., 2017). Herbarium collections and the data they hold are valuable for more traditional studies of taxonomy and systematics, but also for ecology, bioengineering, conservation, food security, and the human social and cultural elements of scientific collection (Culley, 2013; Heberling and Isaac, 2017; Soltis, 2017; Willis et al., 2017). Botanical specimens provide baseline data for basic to applied research applications (Appendix S1; Chapman, 2005). Effects of global change on human health and ecosystem services can be studied using primary biodiversity data, with topics such as the distribution and spread of disease vectors, flora and fauna of economic importance, and the introduction, impact, and spread of non‐native and invasive species (Arnaud et al., 2016; McGeoch et al., 2016).

Significant and irreparable changes to Earth's ecosystems due to global change can be seen by examining the shifts in species distributions and community structure in space and time (IPCC, 2014). By incorporating and combining data sources for environmental factors with biological data, primary biodiversity data from herbaria, and other natural history collections, along with other informative data such as tree ring data, observational records, and phenological and other trait data, analyses can be performed to gain an improved understanding of the impacts of change on global biodiversity. Such research is increasingly requiring collaborative, interdisciplinary science (AIBS, 2015a; Soranno et al., 2015). Botanical data can be used as training data for developing statistical models to predict the way changes will affect organisms. Such models may be used as conservation and policy tools to lessen or mitigate the effects of global change on biodiversity and food security (Jarvis et al., 2008; Guisan et al., 2013). To improve model performance, data gap analysis and focused digitization efforts for particular geographic regions or taxonomic groups may be needed to ascertain data completeness for baseline species distribution assessments (Pino‐Del‐Carpio et al., 2014).

Paleobotanical and paleoecological data, including fossil pollen, stomate size, and evidence of leaf damage by herbivores, can be used to explore species and ecological assemblages over time and against changing environmental parameters (e.g., Strömberg et al., 2013; Kohn et al., 2015; Maguire et al., 2016). Integration of neontological and paleontological biodiversity data, linking with literature‐based occurrence data found in resources such as the Paleobiology Database (PaleoDB; https://paleobiodb.org/) and using application programming interfaces (APIs) and other cyberinfrastructure services such as those becoming available through the enhancing Paleontological and Neontological Data Discovery API project (ePANDDA; https://epandda.org/), is helping to answer deep‐time to present‐day global change research questions. The ability to study communities of organisms through time will require continued coordination of the development of digitization workflows and best practices between collections of different taxonomic groups within both neontological and paleontological collections, with data standardized for efficient integration, aggregation, and downstream use in analyses.

Primary biodiversity data can be used to study changes in communities, temporally and spatially, and shifts in community associations within and between taxonomic groups (Morueta‐Holme et al., 2016). As the volume of biodiversity data from multiple collections of taxonomic and geographic breadth is aggregated, along with supporting observational data records, an assessment of global changes in biological community organization and structure is enabled. Resurveys of biodiversity and the pooling of data across geographic regions, in comparison with legacy data, can be used to assess long‐term shifts in community structure (Verheyen et al., 2017). Long‐term monitoring projects (e.g., the Long Term Ecological Research Network projects [LTER; https://lternet.edu/] and National Ecological Observatory Network [NEON; http://www.neonscience.org/] stations in the United States, the Terrestrial Ecosystem Research Network [TERN; http://www.tern.org.au/] in Australia, and the Chinese Ecological Research Network [CERN http://cnern.cern.ac.cn/en]) are providing open access to long‐term standardized ecological and biological data sets, with the historical data within herbarium collections providing the historical baseline. Phenological data associated with biological collections document changes in seasonality over time and provide insight as to the effect on community associations in a broader context (Davis et al., 2015; Willis et al., 2017).

Species and community assemblages can be indicators of habitat health. Changes in community composition across space and time may be correlated with the appearance of invasive species, changes in environment, or human activity. Baseline documentation of communities as found within botanical collections data sets can be used for restoration or rehabilitation purposes and may be useful for determining surrogate taxa (Weirauch et al., 2017). Collections data sets consisting of both paleological and neontological specimen data are increasingly essential for conservation purposes (e.g., Ponder et al., 2001; Pino‐Del‐Carpio et al., 2014; Barnosky et al., 2017). Organizations such as the International Union for Conservation of Nature (IUCN), World Wide Fund for Nature (also known as the World Wildlife Fund; WWF), The Nature Conservancy, NatureServe, and others benefit from biological collections data and are primarily interested in habitat and species evaluations. Primary biodiversity data are critical for species conservation assessments such as the IUCN Red List (Brummitt et al., 2015) and delineation of protected areas. Biological collections data can be used to provide data for proactive systematic conservation planning or for rehabilitation or restoration efforts, such as the delineation of climate refugia, buffer zones, and corridors. “Alpha” (location of hotspots, design of reserves, restoration assessment) or “beta” (specific species protection, reintroduction programs) conservation questions and policy development can be determined using herbarium voucher specimen data (Soberon et al., 2000). Niche or species distribution modeling using biological collections data can assist with anticipating taxon range shifts, future needs, and restoration parameters due to changes in climatic regimes (Guisan et al., 2013). An example is the Australian‐based Restore and Renew project (https://www.rbgsyd.nsw.gov.au/science/restore-renew), which relies on herbarium records to plan fieldwork for gathering voucher and tissue collections across the entire geographical and ecological distribution of the study species. Surrogate species distributions can also be used to assess rare and endangered species distributions such as the historical and current distribution of communities and host taxa (Morales‐Castilla, 2015; Weirauch et al., 2017). The early detection of incipient invasive species and documentation of the movement and initial invasion point of invasive species depend on primary biodiversity data. Species distribution models can also be used to better understand biological invasions (Guisan et al., 2013) and to identify potential biological control agents (Sutherst, 2014). However, the use of species distribution modeling as a tool for successful conservation planning and policy is often limited by data quality, data availability, and data bias (Cayuela et al., 2009; Elith and Leathwick, 2009). The fitness for use of primary biodiversity data for species distribution modeling (Anderson et al., 2016), agrobiodiversity (Arnaud et al., 2016), and alien and invasive species (McGeoch et al., 2016) has recently been reviewed by Global Biodiversity Information Facility (GBIF) Task Groups. Linkages between specimen collections and conservation information about taxa can be useful for researchers, land managers, policy‐makers, and others interested in protected species or areas. For example, linking specimens of taxa with information about IUCN Red List status, federal or state endangered species listings, or Convention on International Trade in Endangered Species (CITES) restrictions supports research and education.

Linking collections data to phylogenetic data enables the assessment of how global change has influenced or may influence genetic and/or phylogenetic diversity of communities spatially and temporally (Holmes et al., 2016; Soltis, 2017; Allen et al., unpublished manuscript). By linking collections data to different landscape features and assessing how young versus old lineages diversified across space and time, evolutionary trajectories of clades can be analyzed. Biodiversity hotspot analysis can be used to determine regions of interest for further exploration or protection, as well as to supplement the testing of diversity hypotheses and biogeographic theories (e.g., Phillips et al., 2011). Hypotheses of community homogenization, both taxonomic and phylogenetic and including paleontological or pre‐industrial versus modern communities, can be tested.

### Herbarium data fitness for use

Primary biodiversity data, including herbarium data, are not always research‐ready, and the fitness for use of data will depend on the requirements of each research project and the availability and accessibility of information within herbarium collections. Biodiversity data have been described as biased, fuzzy, haphazard, unstandardized, non‐random, incomplete, and unique because of collecting bias and/or digitization gaps, and subsequently require quality assessment (Soberon et al., 2000, 2007; Hortal et al., 2007; Meyer et al., 2015; Gueta and Carmel, 2016; Willis et al., 2017; Daru et al., 2018). Predictive modeling or other statistical analyses may help fill such gaps (Hortal et al., 2007; Chao et al., 2009), but further sampling and digitization efforts are still needed to address spatial, temporal, taxonomic, and data quality gaps and shortcomings (Berendsohn and Seltmann, 2010; Ariño et al., 2016; Troudet et al., 2017).

Taxonomic limitations that users of biodiversity data need to be aware of include the following:

1. Taxonomic or nomenclatural expertise is underestimated for data interpretation (Soberon et al., 2000). Issues associated with taxonomic revision and interpretation are variable across taxonomic groups (Hortal et al., 2007; Troudet et al., 2017).
2. Data aggregators conform to a single synthetic management classification, or taxonomic authority file, for indexing, searching, and discoverability (e.g., the GBIF Backbone Taxonomy), which may result in bias if the raw data are not also considered by researchers (Murray et al., 2017). Lags associated with taxonomic consensus and curation of both physical specimens and data also delay data and updates shared through biodiversity data aggregators (e.g., Bebber et al., 2010).
3. There is often a selective focus on taxonomic groups within collections resulting from the specialty of curators and funded projects (e.g., Daru et al., 2018).
4. Collections often have a selective focus based on taxonomic uniqueness (e.g., endemics), rarity, or economic value. Rare species can often be overrepresented in collections and databases, but are increasingly less represented in more modern collections due to permitting issues. Common species and/or introduced species, by comparison, are often not collected due to the limited capacity and storage of institutions despite the importance of recording current‐day phenological, morphological/anatomical, environmental, or distributional outliers.

Spatial limitations can include the following:

1. Collecting effort is not randomly or regularly distributed (Soberon et al., 2000; Davis et al., 2015), and biodiversity patterns are scale‐dependent and sensitive to spatial resolution (Soberon et al., 2007).
2. Not all specimens have adequate associated locality information, including named locations with distance and direction data, uncertainty information associated with the location, or metadata such as the geodetic datum used to determine latitude and longitude (Chapman and Wieczorek, 2006).
3. Data generalization of sensitive taxa or taxa from sensitive locations can lead to errors in analysis if not documented and detected by users (Chapman and Grafton, 2008).
4. Collections are often focused on nearby accessible geographic regions, such as the proximity to research institution or along roads (Prendergast et al., 1993; Davis et al., 2015; Meyer et al., 2015; Daru et al., 2018), and can be limited in scope due to the regional mission bias of institutions. Intensive localized collection may result from expedition events, ecological assessment, permanent plots or long‐term monitoring, or hotspot analysis (e.g., parks, mountain ranges, wetlands).
5. Geographic collection limitations can be associated with historical, political, funding, or social barriers (e.g., Crawford and Hoagland, 2009).
6. Downscaling information within biodiversity data sets when georeferencing accuracy is low may be problematic for localized analyses.

Temporal limitations can include:

1. Collecting effort is not evenly distributed in time (Soberon et al., 2000; Davis et al., 2015) and has declined since the mid‐20th century (Gardner et al., 2014).
2. Collectors have been, and continue to be, biased in activity during certain time periods both on annual and historical time scales (Prendergast et al., 1993; Daru et al, 2018).
3. Due to the interests of collectors, institutions, and funding agencies, taxa and geographic regions will have temporal biases within collections.
4. Dates may have imprecise month/season/year time ranges, and social and societal differences of date recording (e.g., day/month/year versus month/day/year) may cause confusion in transcription and reduce data fitness for use.

Absence data are important for statistical modeling algorithms. However, such data are rare or not easily discoverable within herbarium data sets. Lack of collections of a taxon at a place and time cannot imply absence. Natural heritage programs and collectors often do capture observational absence information when they search for a taxon, but that information may not be mobilized and is, therefore, effectively unavailable. This is largely a limitation resulting from a data provider's inability to mobilize absence data in standardized fashion, and of the reliability of absence data. The Darwin Core Standard (DwC; http://rs.tdwg.org/dwc/index.htm), the primary international standard for encoding and exchanging specimen and observation data (Wieczorek et al., 2012), has the term ‘occurrenceStatus’ that accepts values of ‘present’ and ‘absent,’ but this standard postdates most collections records. There are DwC terms for sampling effort (‘samplingEffort’), sampling methodology (‘samplingProtocol’), and measurement or fact concept (‘measurementOrFact’), for example, but data are often recorded inconsistently in a comments or remarks text field. Community efforts to develop controlled vocabularies will eventually help to address this issue.

### TDWG data quality standards

Data trust and reliability, even for voucher specimen data, must be evaluated for fitness for use in each research use case (Ariño et al., 2016). There are several community approaches to assess herbarium specimen data quality and biodiversity data in general (e.g., Robertson et al., 2016; Morris et al., 2017). The Biodiversity Information Standards (TDWG) Data Quality Interest Group (DQIG; https://github.com/tdwg/bdq) was established in 2014 with the goals of assessing data quality and assisting with the standardization of the data delivered by aggregators and others. The aims of the DQIG are to establish a framework for “data quality” (Veiga et al., 2017); to standardize how specimen (and observation) records can be evaluated, amended, and reported; and to develop a set of profiles for use cases such as for species distribution modeling. This work focuses on the critical data dimensions of name, space, and time. The work of the DQIG has highlighted the problems associated with the lack of controlled vocabularies within the Darwin Core (DwC) standard. Unconstrained values used among the DwC terms mean that more tests are necessary to detect problems, some data problems cannot be detected, and scientists find it difficult to evaluate the data prior to research use. For example, while five values for dwc:basisOfRecord have been suggested in the standard (e.g., ‘preservedSpecimen,’ ‘humanObservation’), GBIF had (as of June 2017) 2483 distinct values for that term. The outcome of the DQIG's activities will be the development of standard tools for herbarium and natural history collections and record data sets to enable improvement of data quality and fitness for use for a wide range of research questions.

Standard tests being developed by DQIG will be implemented by data collectors for use in the field; by data aggregators such as Integrated Digitized Biocollections (iDigBio; https://www.idigbio.org/), the Atlas of Living Australia (ALA; https://www.ala.org.au), and GBIF (https://www.gbif.org/); by ancillary services such as Kurator (Morris et al., 2017; http://wiki.datakurator.org/); by data users; and by herbarium data custodians. This will provide concise and consistent information for biodiversity data evaluation for different research and data use needs. Most data aggregators currently use test algorithms that report on various potential issues associated with data records, but each aggregator has its own suite of algorithms and reporting methods. Standardization of the tests and resulting assertions, how they are reported, and how these reports as annotations travel with the records are fundamental requirements for efficient research and area management.

### Streamlining field data collection

As technology advances and digital tools are increasingly robust under field conditions, the capture of data in electronic rather than analog format is more efficient and accurate. Such born‐digital data are critical for avoiding further backlog of data transcription in herbarium collections and for efficient downstream incorporation into collection management systems and data aggregators. Digital data capture leads to improved workflows, avoiding errors in transcription and enabling data to be available in a timely manner for global and societal scientific use. Digital technologies and mobile app development allow for locality data (Global Positioning System [GPS]), field images, and other field data elements to be automatically captured and linked, including standardized picklists and vocabularies (e.g., BioCollect; https://www.ala.org.au/biocollect/). One recently developed resource now in use is the Biocode Field Information Management System (FIMS; http://www.biscicol.org). Using this online tool, researchers can develop and customize their data collection protocol, select field headers (terms) from current data standards (e.g., DwC), and then output selected terms in a ready‐to‐use spreadsheet. This system includes the definitions for the terms as well as the data types (e.g., date, text, numeric) expected for each field. In addition, FIMS assigns globally unique identifiers (GUIDs) to each record in the generated template. The FIMS system incorporates several data standards, making it easy for researchers to integrate data. Once the spreadsheets are completed, data can be uploaded via the FIMS validation tool to check data quality and adherence to the expected data standards. The use of QR codes or barcodes with an embedded, computer‐readable GUID equating to a DwC field, such as ‘eventID,’ along with a human‐readable collection number attached to each element of a collection—from the field notebook to the collection tags, to tissues for molecular analysis and images of specimens—assists in the automated linkage and sharing of collections data.

Biodiversity data are collected with a particular use in mind. Additional information beyond the initial application will almost certainly support far broader applications into the future. It is important to recognize that the future of the data can never be fully anticipated, so the collection of additional data and metadata in the field is always a wise investment (Morrison et al., 2017). Even historically, J. Grinnell commented on how the value of collections and the data therein may not be realized in the immediate future (Grinnell, 1910), so he developed and implemented a detailed protocol for recording field observations (Grinnell, 1912).

### Streamlining analysis of aggregated data

The size and scope of aggregated digital data have exploded and will continue to grow with efforts to digitize collections and collect digital biological data directly in the field. Combining large, diverse data sets is currently challenging due to limitations in standards and lack of consistent vocabularies and metadata between research fields. The development of ontologies (e.g., Walls et al., 2014) will help with reducing such barriers between biodiversity resources. However, traditional analysis tools (e.g., spreadsheets, laptops, and databases) have struggled to manipulate the millions of records some research questions require. An approach to addressing this need is to build biodiversity data infrastructures for analyses and not just data aggregation (Poelen et al., 2014). One example is Global Unified Open Data Access (GUODA; http://guoda.bio/), a collaboration between developers and technical staff at Encyclopedia of Life (EOL; http://eol.org), iDigBio, and freelance software engineer Jorrit Poelen. An infrastructure based on Apache Spark (Zaharia et al., 2016) and biodiversity data sets such as EOL, iDigBio, GBIF, and the Biodiversity Heritage Library (BHL) is available for application developers and data analysts to build tools and services providing whole biodiversity data set analytics to explore broad biodiversity questions. As two proofs of concept, EOL and Poelen have developed Fresh Data (http://gimmefreshdata.github.io), a tool to discover and follow records in biodiversity archives that match specific geospatial, temporal, taxonomic, and trait constraints, and Effechecka (http://www.effechecka.org/), in which taxonomic checklists and occurrence lists are returned.

Combining analysis with aggregation of data allows for pattern searching, such as duplicate record resolution, outlier detection of specimen data (e.g., collection outside of collector or environmental range), and batch georeferencing. The work being done by the TDWG DQIG in collaboration with data aggregators to standardize basic data description, output, and data transfer will assist with streamlining such applications.

Digitized images of herbarium specimens for data analysis are increasingly available through biodiversity data aggregators. Aggregated herbarium image data are being utilized for projects such as the automated identification of herbarium specimens (e.g., Carranza‐Rojas et al., 2017; Schuettpelz et al., 2017) and phenological studies (Willis et al., 2017). Historical images available through BHL are becoming increasingly linked to other data sources such as the EOL, ALA, GBIF nodes using the ALA platform, and other sites through community tagging on Flickr (https://www.flickr.com/people/biodivlibrary/). An example is the tagging of images with locality and taxonomic information from *Curtis's Botanical Magazine* (https://www.flickr.com/photos/biodivlibrary/collections/72157681766674633/) for linkage with the herbarium specimens found in ALA.

## Discussion

### Use of research to drive digitization efforts

The Thematic Collections Networks (TCNs) funded through the U.S. National Science Foundation's Advancing Digitization of Biodiversity Collections (ADBC) program provide examples of compelling biodiversity hypotheses to be tested through funded digitization efforts. Novel research hypotheses, geographic and taxonomic themes, and societal demands of health and human services are needed to motivate future digitization and funding, and drive sustainability of collections digitization. GBIF established a task group to address the need to discover biocollections data not yet mobilized (Krishtalka et al., 2016), and others have proposed recommendations to the community and data aggregators for bridging biodiversity data gaps (Berents et al., 2010; Faith et al., 2013; Ariño et al., 2016; Geijzendorffer et al., 2016). Ultimately, the biodiversity data community needs to ask how herbaria, curators and researchers, and policy‐makers should be playing a larger role in driving digitization efforts, and whether recognized data gaps should be preferentially addressed regardless of current research priorities. Improved access to biodiversity portal search data statistics or loan and collection use requests may help support digitization efforts. Including digitization as a component of museum or herbarium accreditation processes (e.g., American Alliance of Museums, National Standards for Australian Museums and Galleries) and strategic planning may help to drive systematic digitization, quality control, and the inventory of botanical collections. This includes encouraging botanical collections worldwide to provide and update information about their institution, holdings, and taxonomic expertise in online resources such as Index Herbariorum (http://sweetgum.nybg.org/science/ih/), the Global Registry of Biodiversity Repositories (GRBio; http://grbio.org/), and iDigBio's U.S. Collections list (https://www.idigbio.org/portal/collections). Ensuring that newly collected data are discoverable and fit for broad reuse requires the community to foster, adopt, and update collection and data gathering best practices and standards through the activities of organizations such as TDWG.

### Education and training needs

Researchers, in particular those early in their careers, need greater exposure to the value of herbarium and biodiversity data available through collections and biological data aggregators. Researchers also need to build skills to be able to interrogate and utilize the available data. Hampton et al. (2017) recently outlined five capstone skills needed by environmental scientists, and by extension, biodiversity scientists and data curators: data management and processing for reproducibility, analysis, software skills, visualization, and communication methods for collaboration and dissemination. An awareness and understanding of the biases, issues, and limitations of the data that are provided are critical for appropriate use of biodiversity data (Gueta and Carmel, 2016; Hampton et al., 2017). Such data literacy and data evaluation skills are needed in the community, from the undergraduate to professional level, for the analysis of large, combined biodiversity data sets (AIBS, 2015b; Hampton et al., 2017). Efforts are underway with the Biodiversity Literacy in Undergraduate Education (BLUE; http://biodiversityliteracy.com) Network, which is developing curricula and building a community network to develop data literacy standards for future research career professionals and the public, who need to be able to interpret the results from global scientific research using botanical and natural history collections data. Reproducible science, appropriate citation, and open data should be priorities in training efforts of the biodiversity community (Bishop and Hank, 2016), with the FAIR Guiding Principles (i.e., Findability, Accessibility, Interoperability, and Reusability) for scientific data management and stewardship as a guiding infrastructure (Wilkinson et al., 2016).

### Born‐digital data and analysis

With technological advances (August et al., 2015), more efficient methods are being developed and shared for the collection of new data (e.g., iDigBio‐sponsored Field to Database [https://www.idigbio.org/wiki/index.php/Field_to_Database] and Georeferencing for Research Use [https://www.idigbio.org/wiki/index.php/Georeferencing_for_Research_Use] workshops). The lessons learnt and the gaps discovered because of the digitization of existing collections need to be carried into future collecting efforts and expeditions. Data curation profiling of biocollections may assist managers and researchers to capture information that informs data curation beyond the technical needs for data ingestion (Bishop and Hank, 2016). Often, collection managers are one step removed from scientists and citizen scientists who have collected or are collecting the specimens. A data curation profile “captures requirements for specific data generated by researchers articulated by the researchers themselves” (Bishop and Hank, 2016), providing metadata that can aid in linking otherwise disparate data sets and making broader reuse of valuable data. Engagement and training of non‐collections personnel, such as environmental scientists and ecologists, is increasingly important for specimen collection and biodiversity data capture (Ward et al., 2015). DNA sequence capture and molecular ecology alone will not resolve the understanding of biodiversity (Creer et al., 2016).

### Internationalization: Engaging and enhancing global digitization

Many large herbaria in the developed world are developing and implementing digitization goals, often with a mandate driven by institutional needs. However, much of the regional diversity and often taxon‐specific collections highly valuable for research are found in smaller local herbaria and museums, which may lack the infrastructure and resources needed to digitize collections (Casas‐Marce et al., 2012). This disparity results in major gaps in primary biodiversity data sets. A critical community goal is to incentivize more institutions to mobilize collections data for physical specimen inventory, curation, and research; this is being done primarily through funding, as well as through training and infrastructural support (Canhos et al., 2015). Biodiversity aggregators are increasingly interested in the use of collections data in research to drive their sustainability, and the physical herbarium collections need data‐use metrics about their collections to maintain funding and institutional support for the continued digitization and publishing of data they transcribe and curate. Appropriate acknowledgment of herbarium collections and their data sets in publications (Rouhan et al., 2017), the use of object and data record identifiers for data tracking (James, 2017), and the development of community standards for citation (e.g., working groups of the Research Data Alliance [Rauber et al., 2015], TDWG Natural Collections Descriptions Interest Group [http://www.tdwg.org/activities/ncd/]) will help to enhance the sustainability of digitization and botanical data mobilization into the future. Appropriate attribution shows advocacy for the continued preservation, expansion, and availability of the physical and digital botanical collections curated by herbaria into the future (Suarez and Tsutsui, 2004; Winston, 2007).
