# Supplementary material for: Herbarium data: Global biodiversity and societal botanical needs for novel research
Source: Appl Plant Sci. 2018 Feb 28;6(2):e1024. doi: 10.1002/aps3.1024 (PMC5851569; doi:10.1002/aps3.1024)
Supplement: Supplementary file 1 [file APS3-6-e1024-s001.docx]

**APPENDIX S1.** Research opportunities using biodiversity specimen-based primary biodiversity data.

1. Species distribution through time and space

1. Species distribution models, species richness models, niche models using paleo- and neontological data
2. Distribution of taxa; range extensions
3. Species rediscovery
4. Endemicity
5. Invasive and non-indigenous species origin, distribution and spread prediction (bioinvasion)
6. Environmental envelopes (patterns of distribution based on environmental conditions)
7. Fossil record to present-day distribution change
8. Anthropogenic impacts (human disturbance, habitat destruction)
9. Community changes (species overlap)
10. Assemblage/community changes through time
11. Reconstruction of distribution; predicted distribution
12. Biogeography; island versus continent
13. Alpha and beta diversity hypothesis testing
14. Relictual distributions

2. Gap analysis

1. Patchy historical collection density (geographic, temporal, or taxonomic) driving focused collecting expeditions
2. Predicting diversity hotspots for focused collecting
3. Collector/specialist changes through time

3. Functional biodiversity

1. Vegetation/habitat models
2. Trait space
3. Anatomical, morphological comparisons, past to present to future
4. Phenotype distributions and changes, paleo- to neontological
5. Phenological changes/shifts; adaptation and evolution; gigantism
6. Ecophysiological characteristics
7. Character-spatial mapping

4. Biodiversity and ecosystems/Earth systems

1. Bioindicators
2. Ecological and paleoecological research
3. Isotopic analyses
4. Ecosystem service models
5. Interaction networks—food webs, pollination, symbiosis, parasitism and herbivory, pathogens, other interactions
6. Evolution of plant–animal (organismal) relationships
7. Economic, human well-being implications (e.g., disease, agriculture, forestry, fisheries)
8. Metagenomics
9. Ground-truthing and training data; carbon assessment
10. Long-term ecological monitoring and inventory
11. Paleoclimate and oceanographic modeling

5. Biodiversity and systematics/taxonomy

1. Species discovery and monographic revision
2. Parataxonomy, automated identification
3. Fossils for dating evolutionary trees
4. Phylogenetic/taxonomic—spatial relationships (phylodiversity) and metagenomics
5. Community assemblages
6. Checklists, floras and field guides, online identification tools, apps
7. Endemism, diversification, and speciation
8. Cryptic species and mimicry complexes

6. Biodiversity and climate

1. Fossils as analog to present day
2. Physiological adaptability (niches)
3. Seasonal community changes (phenology)
4. Identification of bioindicator species/communities

7. Conservation and restoration planning

1. Historical distribution of rare and endangered species
2. Future range prediction based on climate change
3. Urban expansion prediction and species protection
4. Refugia assessment
5. IUCN Red List assessments
6. Population assessment
7. Non-indigenous watch lists and eradication
8. Restoration efforts (e.g., baseline data)
9. Surrogate species (e.g., hosts) for distribution prediction

8. Health and human services

1. Vectors and spread of diseases, non-indigenous taxa
2. Assessment of resource use
3. Agricultural needs and applications
4. Forestry applications
5. Forensic sciences
6. Residential development (e.g., EIS)
7. Fire ecology
8. Historical social linkages (e.g., geneology, humanities)
9. Pesticide drift assessment; pesticide concentrations within tissues or mutation
10. Linking to biochemistry data
11. Linking to National Institutes of Health cancer, toxicology/HIV, etc. screening data
12. Bioprospecting

9. Education and outreach

1. Remote e-identifications
2. Automated identification
3. Biodiversity informatics courses
4. Citizen science applications
5. Policy and decision-making
6. Art and illustration

10. Engineering applications

1. Big data; textual searching
2. Machine learning; neural networks
3. Linking to literature
4. Ground-truthing for remote sensing
5. Bioengineering, bio-inspiration, biomimicry
6. Urban engineering/landscape engineering
7. New data collection tool development

11. Collections management services

1. Data quality, fitness for use, and data cleaning
2. Availability and accessibility of specimens in collections
3. Data gap analysis
4. Data standards improvement and expansion
5. Automated data generation (optical character recognition [OCR], duplicate specimen and record recognition, batch georeferencing)
